# Complete Genome Sequence of the High-Natamycin-Producing Strain *Streptomyces gilvosporeus* F607

**DOI:** 10.1128/genomeA.01402-17

**Published:** 2018-01-04

**Authors:** Gongli Zong, Chuanqing Zhong, Jiafang Fu, Zhilong Zhao, Guangxiang Cao

**Affiliations:** a Shandong Medicinal Biotechnology Centre, Shandong Academy of Medical Sciences, Jinan, China; b School of Municipal and Environmental Engineering, Shandong Jianzhu University, Jinan, China; c School of Pharmacy, Linyi University, Linyi, China

## Abstract

*Streptomyces gilvosporeus* strain F607 is a producer of high levels of natamycin used in the fermentation industry. In this study, the complete genome sequence of strain F607 was determined. This genome sequence provides a basis for understanding natamycin biosynthesis and regulation in a high-natamycin-producing strain and will aid in the development of useful strategies for improving industrial strains.

## GENOME ANNOUNCEMENT

Natamycin (also known as pimaricin), a potent antifungal compound belonging to the polyene antibiotics, has been widely used as a food additive for nearly 50 years and has been generally regarded as safe (GRAS) by the United States Food and Drug Administration. Furthermore, natamycin plays an important role in antifungal therapy, and it was reported to be an effective agent in treating fungal keratitis and bronchopulmonary aspergillosis (1, 2).

Natamycin was originally isolated from *Streptomyces natalensis* (3) and is also produced by a variety of soil-dwelling *Streptomyces* species, such as *Streptomyces gilvosporeus* (4), *Streptomyces chattanoogensis* (5), and *Streptomyces lydicus* (6). The natamycin biosynthesis gene cluster has been partially or entirely sequenced in several streptomycete strains, including in the draft genomes of *S. natalensis*, *S. chattanoogensis*, and *S. lydicus* (7, 8). Studies showed that the natamycin biosynthesis gene clusters in *S. natalensis* and *S. chattanoogensis* have high similarity, and both include five large polyketide synthase (PKS) genes and dozens of genes for tailoring enzymes, transport, and regulation (9, 10). Although the natamycin biosynthesis gene cluster from *S. gilvosporeus* Ins1 has been sequenced (11), no genome sequence of *S. gilvosporeus* has been reported. Due to the considerable commercial value of natamycin, we present here the first complete genome sequence and genomic features of *S. gilvosporeus* F607, a high-natamycin-producing industrial strain, which was developed from ATCC 13326 by various mutagens and collected by our laboratory.

The genome of strain F607 was sequenced using the Illumina HiSeq 4000 platform at the Beijing Genomics Institute (Shenzhen, China). After quality control, about 1,219 Mbp of data were obtained, and about 898 Mbp of data were obtained with the PacBio RSII platform. A total of 8,482,298 bp of genome sequence with an average G+C content of 70.95% was assembled; the genome was predicted to contain one linear chromosome, including 7,145 protein-coding genes, 69 tRNAs, 18 rRNAs (5S, 16S, and 23S), 3 noncoding RNAs (ncRNAs), and 287 pseudogenes. The DNA sequence of the natamycin biosynthesis gene cluster was highly similar to those of the corresponding clusters in *S. natalensis* ATCC 27448, *S. chattanoogensis* L10, and *S. lydicus* A02, with 94%, 93%, and 93% identities, respectively. The gene organization within these clusters and regulatory genes also showed high similarity, except that the *sgnT* gene in strain F607 was on the opposite side of the cluster from the position of its homologues in other species.

In summary, this study presents the first complete genome sequence of *S. gilvosporeus*, which is used in industry due to its high-natamycin-producing ability. Analysis of the genome shows that genes related to natamycin biosynthesis are highly similar to those in *S. natalensis*, *S. chattanoogensis*, and *S. lydicus*. This genomic study will facilitate an understanding of the molecular mechanisms of natamycin biosynthesis and regulation and enable further genetic engineering studies to enhance the production of natamycin.

### Accession number(s).

The genome sequence of *S. gilvosporeus* strain F607 has been deposited at GenBank under the accession number CP020569.
